# Impact of pulse oximetry screening to detect congenital heart defects: 5 years’ experience in a UK regional neonatal unit

**DOI:** 10.1007/s00431-021-04275-w

**Published:** 2021-10-07

**Authors:** Yogen Singh, Si Emma Chen

**Affiliations:** 1 Department of Paediatrics - Neonatology and Paediatric Cardiology, Addenbrooke’s Hospital, NICU, Cambridge University Hospitals NHS Foundation Trust and University of Cambridge School of Clinical Medicine, Box 402, Biomedical Campus, CB2 0QQ Cambridge, UK; 2 Loma Linda University School of Medicine, Loma Linda, CA USA; 3 School of Clinical Medicine, University of Cambridge, Cambridge, UK

**Keywords:** Pulse oximetry screening (POS), Congenital heart defect (CHD), Critical congenital heart defect (CCHD), Screening, New-born infant

## Abstract

Pulse oximetry screening (POS) has been shown to be an effective, non-invasive investigation that can detect up to 50–70% of previously undiagnosed congenital heart defects (CHDs). The aims of this study were to assess the accuracy of POS in detection of CHDs and its impact on clinical practice. All eligible newborn infants born between 1 Jan 2015 and 31 Dec 2019 in a busy regional neonatal unit were included in this prospective observational study. A positive POS was classified as two separate measurements of oxygen saturation < 95%, or a difference of > 2% between pre- and post-ductal circulations. Overall, 23,614 infants had documented POS results. One hundred eighty nine (0.8%) infants had a true positive result: 6 had critical CHDs, 9 serious or significant CHDs, and a further 156/189 (83%) infants had significant non-cardiac conditions. Forty-three infants who had a normal POS were later diagnosed with the following categories of CHDs post-hospital discharge: 1 critical, 15 serious, 20 significant and 7 non-significant CHDs. POS sensitivity for detection of critical CHD was 85.7%, whereas sensitivity was only 33% for detection of major CHDs (critical and serious) needing surgery during infancy; specificity was 99.3%.

*Conclusion*: Pulse oximetry screening showed moderate to high sensitivity in detection of undiagnosed critical CHDs; however, it failed to detect two-third of major CHDs. Our study further emphasises the significance of adopting routine POS to detect critical CHDs in the clinical practice. However, it also highlights the need to develop new, innovative methods, such as perfusion index, to detect other major CHDs missed by current screening tools.

**Table:** 

**What is Known:** *• Pulse oximetry screening is cost effective, acceptable, easy to perform and has moderate sensitivity and high specificity in detection of critical congenital heart defects.* *• Pulse oximetry screening has been implemented many countries including USA for detection of critical congenital heart defects, but it is not currently recommended by the UK National Screening Committee.*	
**What is New:** *• To our knowledge, this is the first study describing postnatal detection and presentation of all the infants with congenital heart defects over a period of 5 years, including those not detected on the pulse oximetry screening, on the clinical practice. * *• It emphasises that further research required to detect critical congenital heart defects and other major CHDs which can be missed on the screening tools currently employed in clinical practice. *

**What is Known:**

*• Pulse oximetry screening is cost effective, acceptable, easy to perform and has moderate sensitivity and high specificity in detection of critical congenital heart defects.*

*• Pulse oximetry screening has been implemented many countries including USA for detection of critical congenital heart defects, but it is not currently recommended by the UK National Screening Committee.*

**What is New:**

*• To our knowledge, this is the first study describing postnatal detection and presentation of all the infants with congenital heart defects over a period of 5 years, including those not detected on the pulse oximetry screening, on the clinical practice. *

*• It emphasises that further research required to detect critical congenital heart defects and other major CHDs which can be missed on the screening tools currently employed in clinical practice.
*

## Introduction

Globally, congenital heart disease, sepsis and lower respiratory tract infections remain the most common causes of neonatal mortality [1]. The incidence of congenital heart defects (CHDs) is around 7–9 per 1000 live births [2, 3]; they account for just under 50% of deaths from all congenital anomalies, and up to 10% of all infant deaths in the Western world [4, 5]. Mortality from CHDs varies from 3 to 7% in industrialised countries to 20% in developing countries [2]. Critical CHDs (CCHDs), comprising up to 30% of all CHDs, are defined by conditions needing surgery, intervention or resulting in death, within 1 month after birth [6]. Most of these defects can be corrected if diagnosed and intervened in a timely fashion; late diagnosis is associated with complications such as acute cardiovascular collapse upon closure of the duct-dependent circulation. Poor clinical condition at the time of surgery furthermore worsens outcomes and mortality [7, 8].

Current screening strategies for detection of CHDs are limited, with a significant proportion of infants with CCHDs remaining undiagnosed before discharge from hospital [9, 10]. Fetal anomaly screening involving antenatal ultrasound at 20 weeks can detect only around 50% of all CHDs [11]; what is more concerning is that CCHDs such as coarctation of the aorta have an antenatal detection rate of just 22% [12]. Post-natal clinical examination to assess heart sounds and inspect for visible cyanosis is similarly poor, detecting only 31% of critical CHDs [13]–[15].

The addition of pulse oximetry screening (POS) can improve detection of CCHDs to around 75–90% [13, 14]. Previous studies have shown POS is accurate, cost effective and acceptable to both parents and clinical staff [5, 13, 14, 15, 16, 17 18]. Pulse oximetry screening is based on the rationale that asymptomatic infants with CHDs will have some degree of hypoxaemia that may not be clinically ascertainable, or a difference in oxygen content between pre-and post-ductal circulations [15] [19]. It can furthermore detect significant hypoxemic non-cardiac conditions, such as sepsis or respiratory conditions [20]. Future utility of pulse oximetry could also be augmented with addition of peripheral perfusion index measurements that may have added value in detection of left-sided obstructive CHDs that may be missed by POS alone [21]–[24]

As a result, routine pulse oximetry screening in neonates has been implemented in many countries globally[5, 25], as well as in around 50% hospitals in the UK despite no recommendation from the UK National Screening Committee (UKNSC)[18, 26, 27]. Many POS studies have been published; however, studies on postnatal detection and presentation of all CHDs, the impact on clinical practice and what cardiac conditions lead to a false negative POS test result are still lacking. The paper is focused on assessing the accuracy of pulse oximetry screening and its impact on clinical practice, including evaluation of postnatally diagnosed CHDs not detected by POS, over a 5-year period at the Rosie Hospital, Cambridge, UK.

## Materials and methods

This was a 5-year prospective observational cohort study involving all 27,170 babies born at the Rosie Hospital between 1 Jan 2015 and 31 Dec 2019. Data were analysed retrospectively. Patients were excluded if they were < 35 weeks of gestation, if they were admitted to NICU before 4 h of age (including babies symptomatic of congenital heart disease), or if they had an antenatal diagnosis of a CHD detected on fetal anomaly screening.

Screening was undertaken routinely by midwives and/or paediatricians on the postnatal ward between 4 and 12 h of age using Masimo’s pulse oximeter sensors with a probe secured with coban tape. Oxygen saturations were taken from the baby’s right hand and right foot to obtain preductal and post ductal saturations respectively. A saturation of < 95% in either pre- or post-ductal circulations, or a difference of > 2% between pre- and post-ductal oxygen saturations was classified as abnormal as per the hospital guideline.

Following an initial abnormal result, infants were assessed — pulse oximetry was repeated 1–2 h later if they were otherwise well with no clinical concerns. The pulse oximetry result was classed as ‘positive’ if the oxygen saturations remained abnormal on a second screen, and these infants were evaluated by a paediatrician for further management. Further clinical details were obtained from the electronic record system, including data on investigations and interventions, respiratory support, infection markers, duration of antibiotics, length of stay and any follow up in outpatient clinics. Babies with false negative POS results were identified from a cardiac database of all babies with congenital heart defects born in the Rosie Hospital.

For the study analysis, CHDs were classified as critical (requiring intervention or resulting in death within 28 days), serious (requiring intervention within 1 year after birth), significant (needing follow up for over 1 year) or non-significant (babies with conditions such as small muscular VSD who had follow-up for less than 12 months). Major CHDs were defined as critical or serious CHDs. Infants with isolated PDA and/or PFO, expected normal findings on echocardiography at this age, were excluded. Definition for the non-cardiac conditions has been summarised in Table 1. The study was approved by the Clinical Audit Department at Cambridge University Hospitals NHS Foundation Trust as per local arrangements for the quality improvement and service evaluation.

**Table 1:** Non-cardiac diagnoses in babies with test positive pulse oximetry

**Condition**	**Definition**	**Number of patients**
**Respiratory distress syndrome (RDS)**	CXR findings, such as ground glass changes, consistent with RDS based on the radiology report	22
**Sepsis**	Raised inflammatory markers (CRP > 10 mg/L) ± positive culture needing antibiotics for ≥ 5 days	126
**Congenital Pneumonia**	Raised inflammatory markers (CRP > 10 mg/L) ± positive culture needing antibiotics for ≥ 5 days and radiological changes on chest x-ray (CXR)	25
**Pneumothorax**	CXR changes consistent with pneumothorax as per radiology report	10
**Meconium aspiration syndrome (MAS)**	History of meconium staining of liquor, respiratory distress, oxygen requirement for longer than 2 h, radiological changes on CXR consistent with MAS	10
**Persistent pulmonary hypertension of the newborn (PPHN)**	Echocardiographic findings consistent with PPHN such as tricuspid regurgitation or flattening or left deviation of the interventricular septum	9
**Transient tachypnoea of the newborn (TTN) requiring oxygen**	Tachypnoea with radiological changes of fluid retention, oxygen requirement for more than 2 h and no rise in inflammatory markers or positive culture	13

## Results

Of the 27,170 infants born during the study period, 25,185 (92.7%) were eligible for pulse oximetry screening, and 1985 (7.3%) were either admitted to NICU before screening or had an antenatal diagnosis of CHD. A total of 23,614 out of 25,185 eligible infants had pulse oximetry screening performed (93.8% uptake). In 1571 eligible infants (6.2%), there were no documented pulse oximetry screening results before discharge from hospital.

**Fig. 1:**
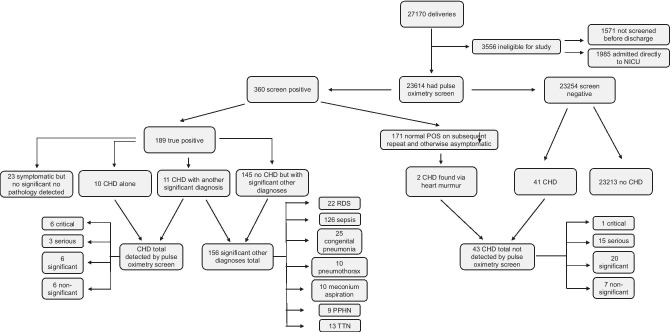
Flow chart showing uptake of pulse oximetry screening and diagnoses of CHDs in infants with positive and negative pulse oximetry results

Of the 23,614 infants who had pulse oximetry screening, 1393 (5.9%) had an abnormal first pulse oximetry screening result. One thousand thirty three of the 1393 infants had a normal second pulse oximetry screening result. Three hundred sixty infants (1.5%) had an abnormal second pulse oximetry screening result and therefore were classified as ‘positive’. As per hospital guideline, these infants were evaluated by the senior or neonatologist, and 171/360 of them were found to have normal oxygen saturation and otherwise clinically well on their assessment. It was the clinician’s decision to classify them as ‘normal on repeat’ in these otherwise well infants. Two of these 171 infants were later noted to have a CHD detected via a heart murmur. The remaining 189 infants (0.8%) had a true positive result as summarised in Fig. 1.

### CHDs in infants with positive pulse oximetry screening test

Of the 189 infants with true positive pulse oximetry screening test, 64 (33.8%) had an echocardiogram performed, and 21 (11.1%) were found to have a previously undiagnosed cardiovascular abnormality (Table 2). Nine infants had a major CHD (6 CCHDs and 3 serious CHDs) while 6 infants had significant CHDs needing follow up for > 12 months after birth.

**Table 2:** Echocardiographic findings and types of CHD in infants with a positive pulse oximetry screening test

**Congenital heart defects in positive pulse oximetry screening cases**			
**Critical CHDs (6)**		**Serious CHDs (3)**	
Hypoplastic left heart syndrome (HLHS)	1	Atrio-ventricular septal defects (AVSD)	3
Hypoplasia of aortic arch	1		
Interrupted aortic arch (IAA)	1		
Coarctation of aorta (CoA)	1		
Critical pulmonary stenosis (PS)	2		
**Significant CHDs (6)**		**Non-significant CHDs (6)**	
ASD alone	1	Small muscular VSD	3
Bicuspid aortic valve with ASD	1	RV hypertrophy	2
Ventricular septal thickening with PFO	1	Tricuspid regurgitation	1
Cardiomyopathy with PDA and PFO	1		
Dysplastic tricuspid valve with PFO	1		
Dextrocardia with PFO	1		

*HLHS* hypoplastic left heart syndrome, *CoA* coarctation of aorta, *IAA* interrupted aortic arch, *PS* pulmonary stenosis, *ASD* atrial septal defect, *VSD* ventricular septal defect, *PDA* patent ductus arteriosus, *PFO* patent foramen of ovale, *RV hypertrophy* right ventricular hypertrophy

In our cohort, CCHD cases included coarctation of the aorta (CoA) (1), hypoplastic left heart syndrome (1), hypoplasia of the aortic arch (1), interruption of the aortic arch (1), and critical pulmonary stenosis (2). Five infants with critical CHD were transferred to a cardiac surgical centre, requiring surgery or medical intervention within 1 month after birth, most of them within a week after birth. One infant with the diagnosis of hypoplastic left heart syndrome went into palliative care after careful discussion with parents who did not wish for them to have any further surgical intervention. All 3 infants with serious category CHDs had surgical intervention within 1 year of age.

All 21 infants with cardiovascular abnormality on initial echocardiography required follow up in the outpatient clinic, and of these, 11 have been discharged at a median age of 409 days (range 7 days–3 years) whilst 10 are still under follow up. One baby with a significant CHD died in this cohort: this was attributed to a genetic TTN13-related cardiomyopathy.

### CHDs in infants with negative pulse oximetry screening test

In total, 43 patients with a postnatal diagnosis CHD had a negative pulse oximetry screen. Twenty one of 43 were found to have a heart murmur on newborn physical examination. Nine of these infants had serious CHDs such as CoA and Tetralogy of Fallot, requiring surgery within the first year of life, whilst 8 had significant CHDs requiring follow up for > 12 months.

Twenty two of 43 infants had negative pulse oximetry screening and a normal physical examination of the newborn. Most of these infants were found to have a heart murmur on routine examination in the community within 8 weeks after birth or had an outpatient echocardiogram arranged for family history of CHD (Table 3). Fortunately, of these 22 infants, only 1 infant had a CCHD. This infant presented in a state of collapse at 12 days after birth and was found to have critical coarctation of aorta and VSD. Six of 22 infants had serious CHD needing surgery within their first year of life, while 12/22 had significant CHDs needing follow up for > 12 months (Table 4). There were no deaths amongst babies with a postnatally diagnosed CHD and negative pulse oximetry screen.

**Table 3:** Presentation of infants with postnatal diagnoses of CHDs and normal pulse oximetry screening test

	**Severity of CHD**				
**Detection**	**Critical**	**Serious**	**Significant**	**Non-significant**	**Total**
Heart murmur detected on newborn physical examination	0	9	8	4	21
Heart murmur after discharge from hospital	0	6	6	1	13
Family history of CHD	0	0	1	2	3
Inpatient echocardiography (for other unrelated cause)	0	0	1	0	1
Pulse irregularity	0	0	1	0	1
Collapse	1	0	0	0	1
Outpatient echocardiography for syndromic screening (T21, William's)	0	0	3	0	3
Total	1	15	20	7	43

**Table 4:** Postnatal diagnoses of CHDs in infants with normal pulse oximetry screening test

**Congenital heart diseases diagnosed in infants with a negative pulse oximetry screen**			
**Critical CHDs (1)**		**Serious CHDs (15)**	
Coarctation of aorta (CoA)	1	Atrio-ventricular septal defects (AVSD)	3
		Partial anomalous pulmonary venous connection (PAPVC) with sinus venous type large ASD	1
		VSD	5
		CoA	1
		Tetralogy of Fallot (TOF)	3
		Dysplastic pulmonary valve with pulmonary stenosis	1
		Aortic stenosis	1
**Significant CHDs (20)**		**Non-significant CHDs (7)**	
ASD	2	Small muscular VSD	7
VSD	8		
Mild pulmonary stenosis	7		
VSD with mild pulmonary stenosis	1		
Aortic arch narrowing	2		

*HLHS* hypoplastic left heart syndrome, *CoA* coarctation of aorta, *IAA* interrupted aortic arch, *PS* pulmonary stenosis, *ASD* atrial septal defect, *VSD* ventricular septal defect, *PDA* patent ductus arteriosus, *PFO* patent foramen of ovale, *TOF* Tetralogy of Fallot, *PAPVC* partial anomalous pulmonary venous connection

### Positive pulse oximetry and significant non-cardiac conditions

One hundred fifty six of 189 neonates (83%), including 11 infants with CHDs, had a significant non-cardiac diagnosis needing further intervention (Table 5). Of these 156, 126 infants required admission to the NICU. Overall, 138/189 (73%) infants required admission to the NICU, including 7/10 infants who had CHDs alone and 5 with transitional circulation (who were admitted for observation).

**Table 5:** Respiratory support and antibiotic therapy in infants with a positive pulse oximetry screening test

**Clinical parameters of babies with a significant non-cardiac diagnosis**				
	**Number of patients**	**Median length (days)**	**Minimum duration (days)**	**Maximum duration (days)**
Mechanical ventilation	7	3	0	6
CPAP/HFNC	80	2	0	18
Low flow oxygen	34	2	0	5
Antibiotics	156	5	2	19
Days of hospital stay		6	2	26

Of the 156 infants with non-cardiac conditions, 103 (66%) patients had a chest X-ray, and 103 (66%) required oxygen therapy during their stay for a median of 2 days (range 1–18 days). Seven (4.5%) infants required mechanical ventilation (median 3 days, range 1–6 days) while 80 (51%) patients required non-invasive respiratory support (continuous positive airway pressure (CPAP) or high flow nasal cannula (HFNC) therapy; median 2 days, range 1–18 days)), and 34 (22%) infants required low flow oxygen therapy (median 2 days, range 1–5 days).

Most infants had more than one significant documented problem (Table 1): 126 had neonatal sepsis (with either a significant rise in inflammatory markers (CRP > 10) or required antibiotics for > 5 days), 7 patients had a positive blood culture, and 25 infants were found to have congenital pneumonia. Ten infants had a small pneumothorax (managed conservatively with no infant requiring a chest drain) while another 10 infants had a diagnosis of meconium aspiration syndrome. Nine infants were noted to have persistent pulmonary hypertension of newborn (PPHN). Twenty two infants were categorised to have respiratory distress syndrome and 13 to have transient tachypnoea of the newborn.

Twenty three of 189 (12%) infants had symptoms of respiratory distress and were given antibiotics for 2 days but had no significant rise in inflammatory markers or any other significant pathology detected.

## Discussion

Globally, many research studies have been performed on pulse oximetry screening for detection of critical CHDs [5, 13]–[15, 19, 20, 25, 28]. Most have focused on the detection of CCHDs and non-cardiac conditions by POS: there remains a paucity of data on missed cases of CCHD and major CHDs not detected by pulse oximetry screening. The biggest strength of our study is that it describes the postnatal diagnoses of all types of CHDs in infants with both positive and negative pulse oximetry screening results over a 5-year period.

In our study of 23,614 newborns, 0.8% had a positive POS result, consistent with previous studies [13, 15]. In total, 64 infants had a postnatal diagnosis of CHDs, including 7 cases of CCHDs. Sensitivity of POS varied from 85.7% for detection of CCHD to just 33% for detection of major (critical and serious) CHD, and specificity was 99.3%. Pulse oximetry screening was able to identify 6/7 (85.7%) cases of CCHDs prior to discharge from hospital. When used in conjunction with physical examination of the newborn, 65.6% of major CHDs were diagnosed prior to discharge from the hospital.

In our study, the post-discharge diagnosis rate of CCHD was 4/100,000. From a retrospective multicentre cohort study of 138,176 infants, Banait et al. [29] reported that the rate of post-discharge diagnosis of CCHDs was almost doubled in infants with no pulse oximetry screening; 7/100,000 in cohorts with POS screening versus 13/100,000 in populations without POS screening (relative risk 0.52, CI 0.2 to 1.42) [29]. However, this difference was not statistically significant which could be because of the small number of CCHDs in the large cohort study. Moreover, there was no significant difference in the mortality at 1 year between the two cohorts as was observed in our study. Similar findings were observed by Campbell et al. [30] who reported that implementation of POS screening had no impact on the post-discharge diagnosis rate of CCHDs or the mortality rate in these infants [30]. However, these were retrospective studies limited to the critical CHDs and did not evaluate the utility of pulse oximetry screening in detecting other clinically significant hypoxaemic conditions (non-cardiac conditions).

Nevertheless, routine pulse oximetry screening is not currently recommended by the UKNSC [18, 27, 31] despite previous studies showing that POS is a low-cost, effective measure [14, 17, 32, 33]. Their argument was that most test positive babies did not have congenital heart disease, yet around half of these test positive babies were diagnosed with other significant clinical conditions such as respiratory problems or infections. In our cohort, pulse oximetry screening similarly detected a large number (156/189) of significant non-cardiac conditions, such as neonatal sepsis, requiring admission to NICU, respiratory support, antibiotics or other interventions. Earlier intervention in these cases is also likely to produce better outcomes in terms of morbidity and mortality, and thus, early diagnosis of these conditions is likewise of importance [13]. Pulse oximetry screening is therefore a valuable tool not only for the detection of CHDs but also for the early diagnosis of hypoxaemic non-cardiac conditions in asymptomatic infants.

There has also been concern that POS may be difficult to implement in district general hospitals where echocardiography cannot be routinely performed and could significantly add to workload. In our study, echocardiography was performed in 33.8% test positive babies, and of these, 32.8% had a cardiac abnormality. Singh et al. [13] reported that echocardiography following a positive POS was more favourable for detection of CHD compared to echocardiography following murmur, with one critical CHD identified per 100 echocardiograms for murmur and one CCHD per 6.8 echocardiograms for pulse oximetry screening [33]. Furthermore, a recent UK survey reported 78% neonatal units felt screening did not increase the number of unnecessary investigations, and 10% felt this increase was justified by the benefits of identifying considerable cardiac pathology[18].

However, as with any screening programme, POS has its limitations. Most importantly, not all CCHDs can be detected with pulse oximetry screening. Despite current screening practices (including POS), timely diagnosis is missed in approximately 900 (15%) infants with CCHD annually in the USA [34, 35]. In our cohort, 1 case (14%) of critical CHD (critical coarctation of aorta) and 15 cases (83%) of serious CHDs had negative POS and were not detected before discharge. A simulation study estimates that of undiagnosed CCHD, 15% would be missed by pulse oximetry screening: acyanotic CHDs such as TOF and CoA are among the conditions most likely to be missed by POS as they do not cause hypoxaemia [27, 28, 30]–[34]. This was similarly demonstrated in our study population, where the major defects missed were TOF, VSD and CoA.

Another limitation of our study was the high false positive rate of 0.7%, compared to the rate of 0.14% in previous studies [5]. This is likely explained by the fact that our screening programme involves performing pulse oximetry screening between 4 and 12 h after birth. It is better to detect critical CHDs as soon as possible after birth, but false positivity is also greatest when pulse oximetry screening is performed < 24 h age: differential saturations may be falsely high < 24 h owing to the high pulmonary artery pressure and patent duct [28, 36]. There remains a challenge in balancing optimal timing of screening with the increasing tendency to discharge apparently healthy babies before 24 h age [28].

### Further research

Our study emphasises on the significance of adding routine pulse oximetry screening to detect CCHDs in clinical practice. However, two-thirds of major CHDs were not detected before discharge. Hence, further research is required to find optimal methods to enhance diagnosis of these missed cases, particularly for acyanotic serious congenital heart defects. Recently, Doshi et al. have published a promising role of adding non-invasive pulse oximetry measurements such as perfusion index (PI), radiofemoral pulse delay (f-hTD) and waveform analysis in improve detection of such cases [21, 22, 24, 37, 38]. In particular, to facilitate earlier detection of left-sided obstructive lesions, perfusion index shows promise as an adjunct to POS, and most modern pulse oximeter models have built-in capability to measure real-time peripheral perfusion [21, 22, 23, 24, 39]. In a Swedish study, all cases of left heart obstructive disease had perfusion index below the interquartile range, with 56% cases below the 5th percentile cut-off of 0.7 [22]. Indeed, combined POS and PI increased sensitivity for detection of systemic critical outflow obstruction to 80% (from 20% with POS alone) [24]. However, there remains a lack of consensus on determining the appropriate cut-off values, and false positive rates are high, although repeat PI measurements with screening at > 12 h may help lower these [40]. Thus, further evaluation of screening protocols in larger trials is required to ascertain the potential clinical impact before its adoption in the routine clinical practice.

## Conclusions

In conjunction with antenatal fetal anomaly screening and physical examination of newborn, pulse oximetry screening can play an important role in early detection of critical congenital heart defects, as well as non-cardiac conditions such as sepsis, pneumonia and other significant pathologies. Our study adds further evidence for implementation of routine pulse oximetry screening to detect critical CHDs. However, there remain concerns that up to 15% of the critical CHDs and a significant proportion of other major CHDs may still be missed prior to discharge from hospital. There is an urgent need of further research in the role of innovative methods such as perfusion index, waveform or artificial intelligence to enhance early detection of these major CHDs that are missed by current screening tools of fetal anomaly screening, newborn physical examination and pulse oximetry screening.

## Data Availability

Any additional data are available with authors and are available upon request.

## Abbreviations

CHD: Congenital heart defects
CCHD: Critical congenital heart defect
CoA: Co-arctation of the aorta
CPAP: Continuous positive airway pressure
CRP: C reactive protein
CXR: Chest X ray
f-hTD: Radiofemoral pulse delay
HFNC: High flow nasal cannula
NICU: Neonatal intensive care unit
PDA: Patent ductus arteriosus
PFO: Patent foramen ovale
PI: Perfusion index
POS: Pulse oximetry screening
PPHN: Persistent pulmonary hypertension of the newborn
RDS: Respiratory distress syndrome
TOF: Tetralogy of Fallot
TTN: Transient tachypnoea of the new-born
UKNSC: UK National Screening Committee
VSD: Ventricular septal defect

## References

[1] Zimmerman MS (2020). Global, regional, and national burden of congenital heart disease, 1990–2017: a systematic analysis for the Global Burden of Disease Study 2017. Lancet Child Adolesc Heal.

[2] Bernier P-L, Stefanescu A, Samoukovic G, Tchervenkov CI (2010). The challenge of congenital heart disease worldwide: epidemiologic and demographic facts. Semin Thorac Cardiovasc Surg Pediatr Card Surg Annu.

[3] van der Linde D (2011). Birth prevalence of congenital heart disease worldwide. J Am Coll Cardiol.

[4] Lozano R (2012). Global and regional mortality from 235 causes of death for 20 age groups in 1990 and 2010: a systematic analysis for the Global Burden of Disease Study 2010. Lancet.

[5] Mn P, Zamora J, Suresh G, Thangaratinam S, Ak E (2018) Pulse oximetry screening for critical congenital heart defects ( Review ) Summary of findings for the main comparison. Cochrane Database Syst Rev (3). 10.1002/14651858.CD011912.pub2.www.cochranelibrary.com. 10.1002/14651858.CD011912.pub2 PMC6494396 29494750

[6] Oster ME, Lee KA, Honein MA, Riehle-Colarusso T, Shin M, Correa A (2013). Temporal trends in survival among infants with critical congenital heart defects. Pediatrics.

[7] Brown KL (2006). Delayed diagnosis of congenital heart disease worsens preoperative condition and outcome of surgery in neonates. Heart.

[8] Fixler DE, Xu P, Nembhard WN, Ethen MK, Canfield MA (2014) Age at referral and mortality from critical congenital heart disease. Pediatrics 134(1). 10.1542/peds.2013-2895. 10.1542/peds.2013-2895 24982105

[9] Wren C, Reinhardt Z, Khawaja K (2008). Twenty-year trends in diagnosis of life-threatening neonatal cardiovascular malformations. Arch Dis Child - Fetal Neonatal Ed.

[10] Abu-Harb M, Hey E, Wren C (1994) Death in infancy from unrecognised congenital heart disease. Arch Dis Child 71(1) 3–7. 10.1136/adc.71.1.3. 10.1136/adc.71.1.3 PMC1029901 8067789

[11] NICOR (2019) National Congenital Heart Disease Audit. 2019 Summ Rep (2017/18 Data)

[12] van Velzen CL, Ket JCF, van de Ven PM, Blom NA, Haak MC (2018). Systematic review and meta-analysis of the performance of second-trimester screening for prenatal detection of congenital heart defects. Int J Gynecol Obstet.

[13] Singh A, Rasiah SV, Ewer AK (2014). The impact of routine predischarge pulse oximetry screening in a regional neonatal unit. Arch Dis Child Fetal Neonatal Ed.

[14] de Granelli AW et al (2009) Impact of pulse oximetry screening on the detection of duct dependent congenital heart disease: a Swedish prospective screening study in 39 821 newborns. BMJ 338(2): a3037–a3037. 10.1136/bmj.a3037. 10.1136/bmj.a3037 PMC2627280 19131383

[15] Ewer AK (2011). Pulse oximetry screening for congenital heart defects in newborn infants (PulseOx): a test accuracy study. Lancet.

[16] Knowles R, Griebsch I, Dezateux C, Brown J, Bull C, Wren C (2005) Newborn screening for congenital heart defects: a systematic review and cost-effectiveness analysis. Health Technol Assess (Rockv) 9(44). 10.3310/hta9440. 10.3310/hta9440 16297355

[17] Ewer AK et al (2012) Pulse oximetry as a screening test for congenital heart defects in newborn infants: a test accuracy study with evaluation of acceptability and cost-effectiveness. Health Technol Assess (Rockv) 16(2). 10.3310/hta16020. 10.3310/hta16020 22284744

[18] Brown S, Liyanage S, Mikrou P, Singh A, Ewer AK (2020). Newborn pulse oximetry screening in the UK: a 2020 survey. Lancet.

[19] Hoke TR (2002). Oxygen saturation as a screening test for critical congenital heart disease: a preliminary study. Pediatr Cardiol.

[20] Movahedian AH, Mosayebi Z, Sagheb S (2016) Evaluation of pulse oximetry in the early detection of cyanotic congenital heart disease in newborns. J Tehran Heart Cent 11(2): 73–78 PMC5027164 27928258

[21] Schena F (2017). Perfusion index and pulse oximetry screening for congenital heart defects. J Pediatr.

[22] de Granelli AW, Östman-Smith I (2007) Noninvasive peripheral perfusion index as a possible tool for screening for critical left heart obstruction. Acta Paediatr 96(10): 1455–1459. 10.1111/j.1651-2227.2007.00439.x. 10.1111/j.1651-2227.2007.00439.x 17727691

[23] Ewer AK (2021). Perfusion index as a screening test for neonatal aortic coarctation: should we be using it routinely?. Acta Paediatr.

[24] Siefkes H (2020). Oxygen saturation and perfusion index-based enhanced critical congenital heart disease screening. Am J Perinatol.

[25] Sola A (2020). CCHD screening implementation efforts in Latin American countries by the Ibero American Society of Neonatology (SIBEN). Int J Neonatal Screen.

[26] Public Health England UK NSC consultation: pulse oximetry as an additional test in the newborn and infant physical exam. https://phescreening.blog.gov.uk/2017/01/10/newborn-pulse-oximetry-screening-pilot-update/

[27] Oddie S, Stenson B, Wyllie J, Ewer AK (2019). UK consultation on pulse oximetry screening for critical congenital heart defects in newborns. Lancet.

[28] Thangaratinam S, Brown K, Zamora J, Khan KS, Ewer AK (2012). Pulse oximetry screening for critical congenital heart defects in asymptomatic newborn babies: a systematic review and meta-analysis. Lancet.

[29] Banait N, Ward-Platt M, Abu-Harb M, Wyllie J, Miller N, Harigopal S (2020). Pulse oximetry screening for critical congenital heart disease: a comparative study of cohorts over 11 years. J Matern Neonatal Med.

[30] Campbell MJ, Quarshie WO, Faerber J, Goldberg DJ, Mascio CE, Blinder JJ (2020). Pulse oximetry screening has not changed timing of diagnosis or mortality of critical congenital heart disease. Pediatr Cardiol.

[31] Evans C (2017) Newborn pulse oximetry screening pilot update

[32] Peterson C, Grosse SD, Oster ME, Olney RS, Cassell CH (2013). Cost-effectiveness of routine screening for critical congenital heart disease in US newborns. Pediatrics.

[33] Peterson C (2013). Hospitalizations, costs, and mortality among infants with critical congenital heart disease: how important is timely detection?, *Birth Defects Res*. Part A Clin Mol Teratol.

[34] Ailes EC, Gilboa SM, Honein MA, Oster ME (2015). Estimated number of infants detected and missed by critical congenital heart defect screening. Pediatrics.

[35] Abouk R, Grosse SD, Ailes EC, Oster ME (2017). Association of US state implementation of newborn screening policies for critical congenital heart disease with early infant cardiac deaths. JAMA - J Am Med Assoc.

[36] Hamilçıkan Ş, Can E (2018). Critical congenital heart disease screening with a pulse oximetry in neonates. J Perinat Med.

[37] Doshi K et al (2020) A novel system to collect dual pulse oximetry data for critical congenital heart disease screening research. J Clin Transl Sci 1–7. 10.1017/cts.2020.550. 10.1017/cts.2020.550 PMC8057385 33948277

[38] Sorensen MW, Sadiq I, Clifford GD, Maher KO, Oster ME (2020). Using pulse oximetry waveforms to detect coarctation of the aorta. Biomed Eng Online.

[39] Piasek CZ, Van Bel F, Sola A (2014). Perfusion index in newborn infants: a noninvasive tool for neonatal monitoring. Acta Paediatr.

[40] Lannering K, Elfvin A, Mellander M (2021). Low false-positive rate of perfusion index as a screening tool for neonatal aortic coarctation. Acta Paediatr Int J Paediatr.

